# Regression with Empirical Variable Selection: Description of a New Method and Application to Ecological Datasets

**DOI:** 10.1371/journal.pone.0034338

**Published:** 2012-03-30

**Authors:** Anne E. Goodenough, Adam G. Hart, Richard Stafford

**Affiliations:** 1 Department of Natural and Social Sciences, University of Gloucestershire, Cheltenham, United Kingdom; 2 Luton Institute of Research in Applied Natural Sciences, University of Bedfordshire, Luton, United Kingdom; Sapienza University of Rome, Italy

## Abstract

Despite recent papers on problems associated with full-model and stepwise regression, their use is still common throughout ecological and environmental disciplines. Alternative approaches, including generating multiple models and comparing them post-hoc using techniques such as Akaike's Information Criterion (AIC), are becoming more popular. However, these are problematic when there are numerous independent variables and interpretation is often difficult when competing models contain many different variables and combinations of variables. Here, we detail a new approach, REVS (Regression with Empirical Variable Selection), which uses all-subsets regression to quantify empirical support for every independent variable. A series of models is created; the first containing the variable with most empirical support, the second containing the first variable and the next most-supported, and so on. The comparatively small number of resultant models (*n* = the number of predictor variables) means that post-hoc comparison is comparatively quick and easy. When tested on a real dataset – habitat and offspring quality in the great tit (*Parus major*) – the optimal REVS model explained more variance (higher R^2^), was more parsimonious (lower AIC), and had greater significance (lower P values), than full, stepwise or all-subsets models; it also had higher predictive accuracy based on split-sample validation. Testing REVS on ten further datasets suggested that this is typical, with R^2^ values being higher than full or stepwise models (mean improvement = 31% and 7%, respectively). Results are ecologically intuitive as even when there are several competing models, they share a set of “core” variables and differ only in presence/absence of one or two additional variables. We conclude that REVS is useful for analysing complex datasets, including those in ecology and environmental disciplines.

## Introduction

Ecological, zoological and environmental research frequently generates datasets comprising one dependent or response variable (*Y*) and multiple independent or predictor variables (*X_1_, X_2_* etc), giving a dataset that is multivariate and multidimensional [1], [2]. Examples include diet and availability of different prey species, animal morphology and climatic variables, disease prevalence and population parameters, and, a particularly common scenario, species-habitat interactions. In the last of these, species abundance, or life-history traits such as longevity, reproductive success, or offspring fitness, are related to multiple habitat variables [3], [4]. As findings from such research are typically used to inform management decisions, it is vital that analyses highlight the most important environmental features (i.e. the causal variables) [5], [6].

### Full model regression

Traditional analysis of such datasets has been based on General Linear Models (GLM), typically using Multiple Linear Regression (MLR). At its simplest, this involves regressing the selected dependent variable against the complete suite of predictor variables. Although this full model regression approach might seem logical, there are several key problems. Firstly, and most importantly, the method does not allow the identification of those factors (if any) that are actually statistically related to the dependent variable [2], [7]. This is against the statistical – and intuitive – concept of parsimony and means that the ecological context often cannot be understood. Consequently, results can be hard to interpret [2], [6], [8] and their potential for informing sensible and sustainable management is reduced [9]. Secondly, having multiple predictors in a model adds noise to the analysis, with the effect that non-significant results may be returned even when the model contains significant predictors (effectively inducing the risk of a Type I error) [10], [11]. Thirdly, multicollinearity can occur within the suite of predictors. Where exact multicollinearity occurs (i.e. when two or more predictor variables are correlated perfectly), there is no unique least squares regression equation and the regression model will fail. Although this is uncommon, approximate multicollinearity (when two or more predictor variables are closely correlated with one another) is common in ecological datasets and renders full least squares regression less robust [12]. These issues mean that parameter estimates from full-model regression are often inaccurate or biased [13].

### Stepwise regression

Because of the problems outlined above, regression processes that involve variable selection have become popular. These seek to identify the “best” subset of predictors and thus simultaneously remove those variables that are redundant: a statistical version of Occam's Razor. Statistically, this means that the noise generated by non-significant predictors is reduced and a parsimonious model is created [14], [15], while biologically this allows understanding of which predictor variables have an important effect on the dependent variable, making the results useful for applied species management. The two main methods are backwards elimination (starting with all predictor variables and removing the least significant first, then the next least significant and so on until all remaining variables are significant) and forward selection (introducing the most significant predictor, then next most significant until all the remaining candidate variables are non-significant) [16]. These approaches can be combined to allow variables to be entered or removed at any stage after the initial step.

The final model of each of a stepwise procedure theoretically comprises the (sub-)set of predictors that have an important effect on the response variable and that best explain the response [17]. However, there are serious issues with this approach [18]. Most importantly, because of the one-at-a-time nature of adding/dropping variables, it is possible to miss the “optimal” model [14], [19]. In other words, although a single variable may be better replaced by a combination of multiple variables, this is not accounted for in standard stepwise algorithms. Indeed, any variable selection model based on the inclusion/exclusion of individual variables without reference to all other variables is likely to be biased [20], [21]. Also, the variable selection typically rests on p-values (but see later), which means that removal of less significant predictors tends to inflate the significance of the remaining predictors artificially [6], [14], potentially leading to type II errors especially when continuous variables excluded from the model are assigned an effect size of zero, despite having some (even minor) effect on the response variable. Contrary to common belief a stepwise approach does not involve calculation and comparison of all possible models. Instead pairs of nested models are compared according to a fixed algorithms, such that only a small fraction of the possible models are actually tested [15], which again means that the optimal model might be missed [22]. Important inconsistencies in selection algorithms can also be overlooked [9]. The number of variables that are entered during stepwise procedures can also cause issues, with final models often having too many variables for reliable interpretation and too few variables for good predictive capabilities [23]. Even the order of the predictor variables in the dataset can affect the selected model, especially when multicollinearity is high [24]. Indeed, because of multicollinearity, a vital variable may not be selected for inclusion because its unique contribution to the model is very slightly lower than the combined power of a subset of other variables entered previously [25]. This is completely counter-intuitive and against the statistical principle of parsimony, which stepwise techniques have theoretically been designed to address. Even in the rare cases where multicollinearity is low, stepwise regression is still likely to lead to locally optimal solutions rather than globally optimal solutions [26]. As a result of all of these issues, different stepwise approaches often fail to converge to the same model as one another, and it can be that none of them converges with the actual optimal model [27].

The main philosophical premise of stepwise regression has also been challenged because it seeks only to identify the Minimum Adequate Model (MAM): the single model that, theoretically, explains the highest amount of variability in the response variable. However, when there are two or more models that have almost equal explanatory power, confidence in a single “final” model could be misplaced [16]. The whole concept of a MAM is also based on the null hypothesis approach to analysis, which itself has frequently been criticised, for example [5], especially for ecological research into species-habitat interactions, which are more descriptive than hypothesis-driven [28].

The statistical and philosophical problems regarding stepwise regression, coupled with the need to optimise the biological meaning of the results obtained and to acknowledge uncertainty, constitute serious weaknesses. These are concerning given the continuing prevalence of stepwise regression in ecological research [9]–[11].

### All-subsets regression

The main alternative to variable-selection regression is all-subsets regression, whereby numerous models are generated – one for each combination of predictor variables – with the best model being selected post-hoc (or, more correctly, the best models to avoid the MAM issues discussed earlier) [21]. Post-hoc selection occurs within an Information Theoretic (IT) Framework and most commonly uses Akiake's Information Criterion (AIC) [29]. When derived for a series of models with the same dependent variable, AIC values can be used to compare those models on using their accuracy (model fit) when balanced with complexity (parsimony). Actual AIC values are inconsequential, rather it is the difference between these values – the so-called delta AICs – that is important (the model with the lowest AIC value, and models that have AIC values close to the minimum, have maximum support – see methods for more details). IT is not based on the null hypothesis approach with arbitrary significance values, but on statistical inference whereby competing models are compared by evaluating their relative support by the data [5], [30], [31].

The all-subsets approach is gradually becoming more common, especially since the publication of seminal papers, for example [9]. The advantages are: (1) reduced reliance on a single MAM, (2) reduced risk that biologically-important variables will be overlooked, and (3) greater confidence in results as the uncertainty of each model can be acknowledged explicitly. However, there are some remaining issues. Where there are few predictors the all-subsets approach is feasible, but with increasing numbers of predictors, the number of models generated increases exponentially. For example, four possible predictors would result in the generation of 15 models, but 30 possible predictors – not uncommon in ecological datasets – would result in 1,073,741,823 models being generated. Undertaking an exhaustive search for the optimal model (i.e. computing, calculating and comparing every possible model) can be done through techniques such as bestglm [6], however, computational time can still be very considerable [26]. Other all-subsets approaches utilise branch-and-bound algorithms to reduce the number of combinations by selecting “pathways” of variable combinations [16]: common techniques are LEAPS and the Gatu method [32], [33], respectively. Although these methods are sometimes considered exhaustive [32], technically they are not totally exhaustive as they do not test every single possible variable combination. Instead, all major “pathways” of variable combinations are tested and pathways with support are investigated more fully. This reduces computational time relative to the exhaustive approach (although the number of models can still be considerable if numerous pathways have considerable support), but the trade-off is that a non-exhaustive search can still result in the optimal model being missed. As a result of this, these subset selection methods may not be satisfactory in terms of prediction accuracy and stability [34] and can frequently miss the optimal model [22]. It should also be noted that even where advances in computer power mean that all-subsets (exhaustive or branch-and-bound) regression is possible, it is not necessarily desirable for such analysis to be undertaken as the large number of models generated means that there is usually support (AIC or equivalent) for a considerable number of competing models. While this is not a problem statistically, it becomes increasingly hard to understand the biological meaning of the models, especially when competing models contain a large number of different variables (as opposed to minor differences or different combinations of similar variables). This was exhibited by work by on bird-habitat associations, when 42 highly-supported competing models, with many different variable combinations, were generated [9].

### Remaining problems and the need for a new approach

There have been attempts to combine the statistical advantages of AIC and the intuitive appeal of stepwise procedures [23] with AIC values being used instead of p-values as a method of variable selection in stepwise regression. In this approach, a decrease in AIC of at least 2 is needed for an extra parameter to be added in forward-selection or retained in backwards-selection (this is the minimum for any improvement in model fit not to be outweighed by the addition of an extra parameter:[18]. The issues with this are: (1) the continuing use of a one-at-a-time method of adding/dropping variables with an arbitrary cut-off with all the inherent weaknesses described above, (2) the continuing focus on MAMs, and (3) the fact that when used as a method of variable selection AIC will always enter predictors from a set of independent variables, even when none are significantly related (i.e. at least one model will always be generated) [35]. Moreover, in a backwards selection scenario in ecological datasets, the elimination of variables may stop at a given point because no single variable will reduce AIC sufficiently. However, if elimination of the weakest variable is forced manually at this step, substantially lower AIC values can result at later stages (*pers. obs.*). Another approach has involved development of the closely-related, piecewise linear, regression shrinkage techniques LASSO [36] and least angle regression (LARS) [37]. These work in a similar way to stepwise regression but, rather than adding/subtracting variables *per se*, they instead alter predictor coefficients in the direction their correlation with *Y*. These approaches can be used effectively in some cases, for example [38], but are often over complex (i.e. not maximally parsimonious) and are still very vulnerable to error when there is high multicollinearity [26].

The need for new regression techniques to circumvent some of the issues discussed above has been repeatedly highlighted in statistical literature [2]. More specifically, there have been calls for development of a robust method of variable selection (based on the empirical support for each variable rather than computer algorithms) to create a few competing models that have optimum prediction performance and that can be compared post-hoc using AIC [39]. In this paper, we detail a new technique that combines IT and traditional linear regression approaches to analyse complex ecological datasets. In this new method, which we have named REVS (Regression with Empirical Variable Selection), we combine the rigour of all-subsets regression, the convenience and intuitiveness of stepwise procedures, and the transparency of post-hoc multiple model consideration. This demonstrates a ‘thinking approach’ to analysis [40]. We test the effectiveness of the new technique in relation to full and stepwise regression using a case study dataset relating nest site parameters to avian offspring fitness, and verify it using a further 10 exemplar datasets. The R code for REVS is provided as supplementary material.

## Methods

**Figure 1:**
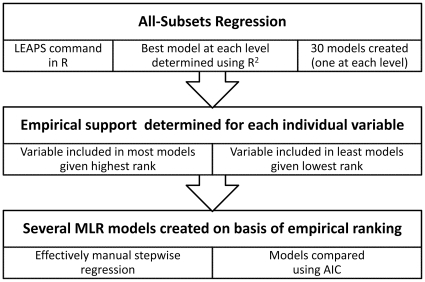
The REVs procedure outlined (MLR = Multiple Linear Regression).

The basic premise of REVS is to use branch-and-bound all-subsets regression to quantify the amount of empirical support for each individual variable in the dataset. A series of regression models is then created; the first model containing only the variable with the most empirical support, the second containing this variable and the one with the next most empirical support and so on. Full regression models are created at each step, which can then be compared post-hoc. If it is desirable to have a final Minimum Adequate Model (MAM), the model with the highest R^2^ and lowest P value is indicated. If, as is usually the case, it is better to consider multiple models, the models can be compared using delta AIC values. Full details of each process, and their rationale, is given below, and outlined in Fig. 1. All processes are linked within the single REVS code, which is supplied here as Supporting Information (File S1), together with a sample dataset (File S2) and a short ReadMe document (File S3). This last is intended to supplement those given below by providing a practical introduction to REVS; it also describes how to run REVS on the sample data (File S2) and briefly interprets the results obtained by so-doing.

### All subset regression stage

All-subsets regression is run on a given dataset within REVS using the R library LEAPS [8]. This employs sophisticated branch-and-bound techniques to run combinations of variables within each level; a level being defined as the number of variables allowed into a given model at any one time [32]. In REVS, LEAPS is parameterised to output the best model at each level (i.e. the best model containing one variable, the best model containing any two variables etc.). The best model at each level is determined by comparison of the R^2^ values of the candidate models; the model with the highest R^2^ value being selected. The number of levels, and thus the number of models, equals the number of predictor variables in the dataset (e.g. if there are 25 independent variables, there are 25 levels (level one having one variable, level two having two variables and so on) and thus 25 models (one at each level)). When LEAPS is run independently of REVS, the output is a square matrix with individual variables as columns and levels as rows (the matrix is square by virtue of the fact that the number of variables will always equal the number of levels – see above). If the value of an individual cell of any variable/level combination is TRUE, the specified variable has been included in the best model at that level, otherwise FALSE is returned. Thus, by counting the number of TRUE values for each variable, an empirical ranking value can be accorded to that variable (e.g. if a variable has been included in 20 out of 25 models, it gains an empirical ranking of 20). Theoretically, a single ranking value will be accorded to each variable between 1 (the variable with least support; only being included in the final model that enters all variables) and *k* (*k* being equal to the actual number of predictors; which would be accorded to the variable with the most support by virtue of its inclusion in every model). In practice, however, this does not always happen as there can be ties (for example, two variables each being included in 10 models each). This might happen, for example, when one variable is superior at Level 1 (where one variable is entered) but not needed at Level 2 (where two different variables are better). It should be noted that when LEAPS is run within REVS, the TRUE/FALSE matrix is not displayed, but the empirical ranking is calculated and stored. Because of limitations in the LEAPS function, the maximum number of variables that can be included in a single dataset is 32 (31 predictors and 1 dependent variable).

### Model generation stage

Once the empirical support for each variable has been ascertained in the form of a ranking, a series of regression models is created. The first model contains only the variable with the most empirical support, the second contains this variable and the one with the next most empirical support and so on. In essence, this is similar to a manual stepwise process, but differs in that the entry order of variables is based on empirical evidence not an algorithm. It addition to detailing which variables are in each model, traditional estimates of model fit (adjusted R^2^) and significance (P) are given, along with AIC values and delta AIC values to allow multiple models to be compared in an IT framework. As noted above, AIC values are based upon combining model fit (based on log-likelihood, which is related to the Kullback–Leibler distance) and parsimony (the number of explanatory variables in the model, *K*) using the formula AIC = −2(log-likelihood)+2*K*. High AIC values suggest comparatively poor model fit, but this is not intuitive. Thus, delta AIC values are calculated for each model to show the amount of support (Δ) for a given model (*i*) using the formula Δ_i_ = AIC_i_−AIC_min_ (AIC_min_ is defined as the AIC value of the model that has the lowest AIC score from a series of competing models). Models that have low Δ values can be considered superior to those with high Δ values using a relative scoring system [21]: Δ values of 0–2 = very strong support; 3–4 = strong support; 5–9 = considerably less support; >10 = essentially no support.

We strongly suggest that when comparing between models, delta AIC values are used rather than R^2^ values. This is because although R^2^ gives a simple measure of model fit by quantifying the amount of variability in the dependent variable explained by predictors, the addition of any additional variable (or even random noise) into a model will increase this value. Although this is allowed for in REVS (and many other MLR approaches) by use of the adjusted R^2^ value so that there is no spurious increase in model fit estimation, this does not actually penalise the model on the basis of parsimony. Conversely, AIC calculation uses both model fit and parsimony to reduce the chance of over-complicated models being supported when a less complicated model is almost as good [30]. R^2^ values do, however, provide a useful and intuitive measure of model fit in the selected models, hence their retention in the REVS output.

Sometimes, the empirical ranking will contain ties for reasons explained above. When this occurs, REVS will, by default, enter individual variables within a tie in the order in which they appear in the datasheet. Such ties, and their arbitrary order of entry, do not matter if all tied variables are entered into the best (lowest AIC) model. For example, if there is a tie between variables (e.g. 3a and 3b) and both are entered into the best model (together with other variables if appropriate) this will not bias results. However, it is possible that one of a pair of variables (e.g. 3a) is entered and the resultant model is considered the best model before the other tied variable (3b) is entered, because parsimony outweighs a relatively small increase in model fit. In this case, it remains possible that a better model would have been generated if the alternative variable had been entered instead. To circumvent this, where there are ties in the dataset AND the best model is generated without both tied variables being entered (1, 2, 3a), the alternative is also created (1, 2, 3b). The better model is then selected on the basis of delta AIC. In the case of a 3-way tie (e.g. 3a, 3b, and 3c), all options are tested. REVS allows for up to a five-way tie (i.e. equal empirical support for up to five variables). Ties between a greater number of variables than this are extremely unlikely because the number of predictors is constrained (indeed, extensive testing has failed to find an occasion where are dataset generated anything higher than a four-way tie).

### Rationale

The advantage of using all-subsets regression to rank support for candidate predictor variables empirically is four-fold. Firstly, it avoids the one-at-a-time method of adding or dropping variables that is so problematic in stepwise regression [14]. It allows for, and quantifies, situations where a specific variable is best at Level 1 but where two different predictors are better in tandem at Level 2 and three totally new variables are better at Level 3; something that current stepwise algorithms cannot achieve. As such, the best model at each level is independent of all others. This is particularly important when multicollinearity is high [12], as it is in many complex ecological datasets. Secondly, ties are explicitly allowed for, such that the order that the variables are listed in the dataset does not matter, as it can do in conventional stepwise regression [24]. Thirdly, because adjusted R^2^ values are used during the all-subsets analysis, there is no demarcation of variables as being “important” or “unimportant” of the basis of an arbitrary significance value. The situation whereby removal of less significant predictors tends to inflate the significance of the remaining predictors artificially [14] is also avoided. Fourthly, and most importantly, the order of entry into what is essentially a manual stepwise procedure is based on empirical evidence rather than an algorithm that can change even between software packages [9]. Thus the process is completely transparent with no masking of analytical uncertainties.

The empirical ranking of each variable provides the researcher with some understanding of the importance of individual parameters. There are, however, several reasons for using this information as a means to an end rather than the end itself. Constructing new models based upon empirical ranking means: (1) model fit can be ascertained; (2) models can be used predicatively; and (3) AIC values are generated to allow comparison of multiple models. Moreover, without building new models, it would be unclear what the demarcation between a variable being an “important” or “unimportant” influence on the dependent variable should be. Any rule-of-thumb figure, such as a variable being regarded as “important” if it is included in more than 50% of models, would be without empirical support and akin to using an arbitrary significance value [15].

When compared to all-subsets approaches, REVS may also be superior. It requires much less computing power than exhaustive all-subset approaches (which is particularly important in cases when there are numerous independent variables as computational time increases exponentially) and can be more robust than sole use of branch-and-bound all-subset approaches, such as LEAPS. To expand on this last point, because REVS utilises LEAPS to quantify empirical support for each variable in a given dataset, theoretically, the best REVS model and the best LEAPS model should be identical. However, because LEAPS does not perform an exhaustive search for the “best” model from a series of predictors, the optimal model could be missed. Although the REVS approach takes information from LEAPS (the empirical support for each variable), it does not rely on LEAPS isolating the optimal model. Instead, a new model is created based on the empirical support rankings, which is akin to an incremental search for the lowest delta AIC (and thus the optimal model). Moreover, contrary to all-subsets regression, REVS is variable-driven rather than model-driven. In LEAPS, or any other type of all-subsets regression, variables in the best model may be very different to those in the second best model and so on. This is logical statistically, especially when there is multicollinearity in the predictor variables, but confusing both biologically and from an applied perspective, especially when there are several competing models with a delta AIC<2 (since these are treated as equivalents under traditional use of AIC). REVS avoids this by first determining how much support is there for each variable (using the all-subsets matrix) and then determining where the cut-off point is for variables to be useful in predicting the dependent variable (additional of variables stepwise until the model does not substantially improve). This is easier to understand as, even if there are two or more competing models within a delta AIC of <2, they will have many variables in common and only differ in whether or not additional variables are added (i.e. the main “core” is the same and there are just minor differences in presence/absence of additional variables). This makes interpretation much easier than comparing a series of models that are fundamentally different – a situation that often applies in ecology [9].

### Case study dataset

The condition of altricial birds when they fledge from their natal nest is a key determinant of their immediate and first-winter survival [41], [42], longevity [43], recruitment into the breeding population [44] and future reproductive success [45]. The influence of habitat variables on fledging condition is therefore important to deepen understanding of environmental influences on life-history parameters. Although condition is difficult to measure directly, wing length (the best univariate size predictor) is an excellent proxy [46].

Wing lengths of juvenile great tits (*Parus major*) raised in nestboxes at Nagshead Nature Reserve (Gloucestershire, UK) were recorded by AEG under licence from Natural England (Number 20060590) in 2006. In total, measurements were taken of 232 chicks in 50 nests. All measurements were taken 15 days post-hatching, when wing lengths were fully grown [47]. Mean wing length was calculated for each brood to avoid pseudoreplication; and this constituted our dependent variable, *Y*. Concurrent fieldwork was undertaken to quantify the habitat surrounding each nest. Vegetation data giving information on species and structure (Table 1) were collected from circular nestbox plots, which were 11.3 m diameter and provided a survey area of 100 m^2^ (0.01 ha) centered on the nestbox tree [48]. In addition, distances from each nestbox tree to the nearest permanent water source, footpath, vehicular forest track (used by forestry workers and reserve staff) and main roads were quantified by comparing GPS location of each nestbox to all water, path, track and road datapoints using Pythagoras' theorem and selecting the lowest value for each parameter. In total, there were 25 independent variables. All necessary permits were obtained for the described field studies; specifically bird handling was undertaken under licence from Natural England (Number 20060590; licensee AEG) and no specific permissions were required for collection of the habitat data since work was entirely survey based (i.e. non-manipulative). Permission to work at Nagshead Nature Reserve was provided by the Royal Society for the Protection of Birds (RSPB) and Natural England.

**Table 1:** Vegetation variables in the case study dataset.

Generic habitat variables	Specific habitat variables
Number of trees	Number of Pedunculate oak (*Quercus robur*)
Distance to the nearest tree	Number of silver birch (*Betula pendula*)
Number of saplings	Number of beech (*Fagus sylvatica*)
Number of shrubs	Number of rowan (*Sorbus aucuparia*)
Percentage of ground cover	Number of sycamore (*Acer pseudoplatanus*)
Diversity of trees	Number of white/downy birch (*Betula pubescens*)
Diversity of saplings	Percentage coverage by holly (*Ilex aquifolium*)
Diversity of field-layer species	Percentage coverage by hawthorn (*Crataegus monogyna*)
Total plot diversity	Percentage coverage by bramble (*Rubus fruticosus* agg.)
Grazing regime (grazed or not)	Percentage coverage by bracken (*Pteridium aquilinum*)

Previous research [49] analysed the effect of nestbox orientation on wing length, but the potential influence of the other variables was not considered, making this an ideal (and ecologically typically) dataset on which to trial REVS. To assess the effectiveness of the REVS technique, the same case study dataset was also analysed using full model regression, stepwise regression, and LEAPS all-subset regression using AIC and R^2^ values. Given that calculating goodness-of-fit statistics, such as R^2^, can cause inflation in apparent model fit [50], [51], comparing methods using spilt-sample cross-validation is preferable when sample sizes permit [1], [19]. Accordingly, we performed a validation where we removed 10 cases from our original dataset at random to create a hold-out dataset and then calculated REVS, the full regression model, the best stepwise model, and the best LEAPS all-subset model using the remaining 40 cases. On a per-model basis, we then used gradients and intercept information to calculate the predicted value of *Y* (great tit wing length) for the 10 cases in the hold out sample. Using a separate regression analysis for each of the four models, predicted values of *Y* (*Y_predicted_*) were compared with actual values of *Y* (*Y_actual_*) on the basis of R^2^ values (higher values = better prediction accuracy) and residual sum of squares (RSS; low figures = low error) as honest model fit estimates and honest error rate predictions, respectively [26].

### Additional datasets

In addition to the detailed consideration of the above dataset, we also tested REVS on a further 10 ecological datasets to establish the general applicability of the technique as a model fitting process as per [6]. Half of these additional datasets were concerned with species-habitat relationships (mammals and birds); the remaining datasets were more general, covering plant morphology, animal behaviour, human biology, microbiology and environmental biology. All datasets are detailed briefly in Table 2 and were either previously published (and details of ethical considerations can be found therein), or were unpublished collected without the need for specific permits. Again, datasets were also analysed using full, best stepwise and best LEAPS all-subset regression models by comparing model-specific AIC values [14], [52]–[54].

**Table 2:** Details of the 10 additional datasets (the top five datasets are on species-habitat interactions; the second five datasets are wider biological datasets).

Dataset details	Dependent variable	Independent variables	Cases	Source
Blue tit nest site selection (Nagshead, Gloucestershire)	Frequency of nestbox occupations over (15 years)	20 nestbox variables (e.g. size, height, location)	295	A Goodenough; unpublished data
Great tit nest site selection (Nagshead, Gloucestershire)	As above	As above	295	A Goodenough; unpublished data
Dormouse nest site selection (Midger Wood, Gloucestershire)	Frequency of nest tubes occupation (13 years)	25 variables describing surrounding habitat	100	R. Williams; unpublished data
Pied flycatcher clutch size (Nagshead, Gloucestershire)	Mean number of eggs per clutch per nestbox (15 years)	31 variables describing surrounding habitat	258	[4]
Pied flycatcher fledging success (Nagshead, Gloucestershire)	Mean number of fledglings per brood per nestbox (15 years)	As above	254	[4]
Plant morphology (Lady Park Wood, Gwent)	Canopy coverage	4 tree-specific variables, including height and DBH	300	A Goodenough; unpublished data
Animal behaviour	Average time spent in slow wave sleep per 24 hours	7 life-history variables (e.g. weight, gestation, lifespan)	62	[55] Available from: http://lib.stat.cmu.edu/datasets/sleep]
Human biometrics	Percentage body fat (underwater weighing)	14 measurements (e.g. weight, height, chest circumference)	252	Data from R. Johnson; available from: http://lib.stat.cmu.edu/datasets/bodyfat
Aquatic bacterial load (River Severn, Gloucestershire)	Total bacteria plate count from 100 µl water on nutrient agar	5 chemical parameters (nitrogen, calcium, pH etc)	12	S. Eley; unpublished data
Organic pollution (Oslo, Norway)	Amount of organic particulate matter (log transformed)	7 environment parameters (e.g. wind speed, time of day)	500	Data from M. Aldrin; available from http://lib.stat.cmu.edu/datasets/PM

Running the REVS procedure on these datasets took <1 min.

## Results

### Case study dataset

Analysis with REVS gave much higher R^2^ values, considerably lower delta AIC values and lower p-values than were produced with full or stepwise regression models (Table 3). The full model was non-significant, despite containing significant predictors, and had a very high AIC value that was within the “no support” category of >10 [21]. Three models were created during the stepwise process; adding, in order, orientation category, percentage cover bracken and percentage ground cover. In total, 25 models were created using REVS (as there were 25 independent variables) and the best model on the basis of delta AIC and R^2^ entered eight predictors (Table 4). The best LEAPS all-subset model, contained five predictors, of which two (orientation category and percentage cover bracken) were included in both the best stepwise model and the best REVS model. REVS was superior to LEAPS (higher R^2^), although the delta AIC distance of both models was <2, such that they both had considerable support.

**Table 3:** Comparison of REVS against full model regression, stepwise regression (*P* to enter = 0.05) and LEAPS all-subset regression for the case study dataset of great tit chick fitness (quantified using wing length) as the dependent variable and 25 independent habitat parameters.

Model	Complete model (*n* = 50)				Comparison of *Y_predicted_* with *Y_actual_* for 10 cases in a hold-out sample	
	Adjusted R^2^	AIC	Delta AIC	P	Adjusted R^2^	RSS
REVS (best model^1^)	0.374	156.00	0.00	0.0005	0.478	62.745
LEAPS all-subsets (best model^2^)	0.331	157.40	1.40	0.0007	0.104	353.713
Stepwise (best model^3^)	0.254	160.71	4.71	0.0014	0.439	80.229
Full	0.034	184.74	28.74	0.4899	0.449	76.986

1 For variables included in the best model, see Table 4.

2 Variables included: orientation category (−), percentage cover bracken (+), percentage cover holly (−), diversity of field-layer species (+), and canopy coverage (+).

3 Variables included: orientation category (−), percentage cover bracken (+), and overall percentage cover ground (−).

The full models are detailed and the prediction accuracy of each is calculated using a hold-out sample (see methods for more details).

**Table 4:** REVS models for analysis of great tit wing length giving R^2^ and delta AIC values.

Model	Adjusted R^2^	Delta AIC
Orientation category (1 = S-SW; 0 = other; negative relationship)	0.074	14.072
+Distance to nearest path (positive relationship)	0.085	12.535
+Number of trees (positive relationship)	0.165	9.920
+Number of silver birch (positive relationship)	0.258	5.011
+Distance to nearest water source (negative relationship)	0.314	2.066
+Percentage of ground cover (positive relationship)	0.318	2.596
+Number of downy birch (negative relationship)	0.342	1.681
**+Number of saplings** (negative relationship)	**0.374**	**0.000**
+Distance to nearest road (positive relationship)	0.369	1.191
+Percentage holly coverage (negative relationship)	0.355	2.970

Each row shows the latest variable to be entered into the model (in addition to those previously added) and the overall adjusted R^2^ and delta AIC. The model in bold was the single best model when models were compared using delta AIC (or R^2^). All models were significant (*P*<0.05).

Recalculation of the models on subset of the data (*n* = 40 cases) allowed quantification of honest R^2^ and honest error rate through comparison of *Y_predicted_* with *Y_actual_* for the 10 cases in the hold-out sample. This showed that the REVS model was superior (higher R^2^, lower RSS) to all other models (Table 3). The low predictive accuracy (and low honest R^2^/high RSS) of the LEAPS all-subset model was surprising, but partially explained by substantial discord between *Y_predicted_* and *Y_actual_* for one case using this model (if this case was excluded, R^2^ rose to 0.238 and the RSS decreased to 150.728).

When considering the actual variables entered in the different models, two things are worthy of note. Firstly, there were substantial differences in the variables included in the best REVS model compared to the best stepwise and best LEAPS all-subset models. The best LEAPS model included percentage cover holly, diversity of field-layer species, and canopy coverage; all variables that were absent from the best REVS model. Moreover, one of the three factors (percentage cover bracken) that was included in both the best stepwise and best LEAPS model was not included in the best REVS model – indeed this variable was ranked 20 out of 25 using this process. The fact that it was included in the stepwise/LEAPS models, when it actually did not correlate univariately with wing length as the response variable, was possibly because it correlated significantly (*P* = 0.002) with the distance to the nearest path (the second ranked variable using REVS: Table 4). Secondly, it is worth noting that, univariately, the number of trees in the nestbox plot, which was entered into the best REVS model, did not correlate with great tit wing length (*P* = 0.988), and so would never have been included in a “one-at-a-time” stepwise model, and would probably always be ignored by branch-and-bound all-subsets approach. However, this variable is key in combination with other variables (being included in 23 out of 25 models created using REVS, making it the third most common parameter).

### Additional datasets

**Figure 2:**
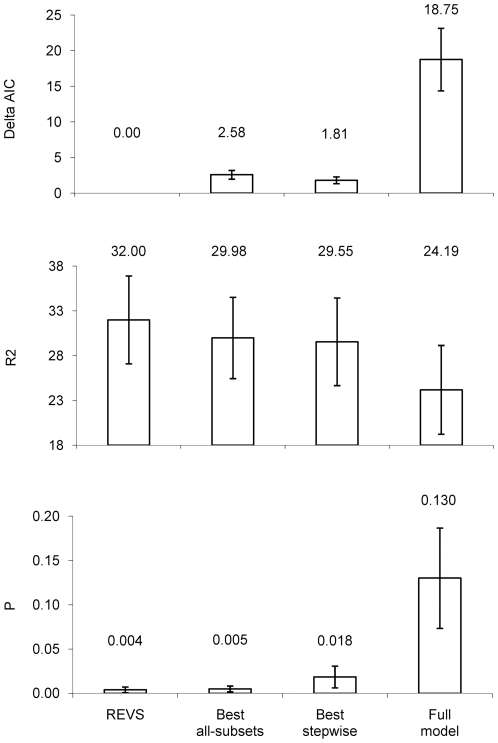
Mean (± se) results of running 10 datasets (detailed in **Table 2**) through REVS compared with standard full regression and stepwise regression for (a) delta AIC values (combines model fit and parsimony; lower values are preferable); (b) R2 values (higher values are preferable) and (c) significance (P values; lower values are preferable). It should be noted that the delta AIC value for REVS was 0 in all cases.

In terms of delta AIC values, significance and R^2^, the REVS model was superior to full models for all 10 datasets tested, superior to stepwise regression for 8/10 datasets (in the remaining datasets, the best REVS model was exactly the same as the best stepwise model with the same model fit and significance values), and superior to LEAPS all-subsets regression in 4/10 datasets (again in the remaining datasets, the best REVS model was exactly the same as the best LEAPS all-subsets model). Crucially, REVS was never worse than any other method. On average, delta AIC was 0 for the best REVS model compared with almost 20 for the full model (Fig. 2a) while mean R^2^ values were higher for models generated using REVS compared to any of the traditional MLR methods (Fig. 2b). Overall, R^2^ was 32% higher when REVS was used in place of the full model and 8% higher when REVS was used rather than stepwise or LEAPS all-subset regression. P values for REVS were, on average, much lower than for full or stepwise models, and very similar to LEAPS (Fig. 2c).

**Figure 3:**
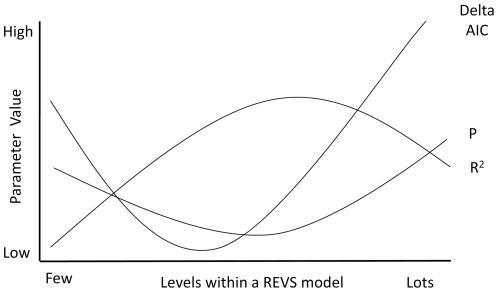
Conceptual graph showing how REVS regression parameters (delta AIC; R2 and significance) relate to one another and how they change at different levels for the same dataset (a new independent variable is added at each level; thus giving a more complex model). This is based on running 10 sample datasets (Table 2) though REVS.

The complex relationship between delta AIC, R^2^, and significance is shown in Fig. 3. Generally, when comparing the different REVS models for each dataset, generally the AIC value was at its optimum (lowest) with a slightly more conservative model (i.e. fewer levels and thus with fewer independent variables) compared to the optimum (highest) R^2^ value (Fig. 3). This was expected because while adjusted R^2^ values allow for the inclusion of an additional variable into the model to avoid non-helpful predictors inflating the variance explained artificially, they do not penalise the model for the inclusion of an additional variable. Thus, unlike AIC, they do not reduce the chance of over-complicated models being supported when a less complicated model is almost as good [30]. The lowest significance value using REVS usually occurred at the same level of model complexity as the highest R^2^ value, but was occasionally better one level higher (i.e. better if one more variable was added to the model) (Fig. 3).

### Intuitive interpretation

As noted in the methods, REVS is variable-driven rather than model-driven. Thus, even when two or more competing models have a delta AIC<2 (i.e. essentially equal support), they will have the same “core” set of variables, whereas two competing all-subsets models might be very different. For example, in the case study dataset, seven models were generated with LEAPS that had a delta AIC<2, but they differed substantially. Nine variables were entered into the top three models combined, but only three of these (orientation, diversity of field-layer species and percentage bracken) were common to all three models, with the rest typically appearing in just one of the three. A similar situation occurred for one of the additional datasets on body fat (see Table 2), where 7 of the 13 models LEAPS generated had a delta AIC<2: four variables occurred in each of these in different combinations with a further seven variables. Conversely, when REVS was used, interpretation was simplified. Firstly, there were fewer competing models with delta AIC<2 (three and five for the great tit and body fat datasets, respectively), which meant that fewer comparisons were necessary. Secondly, and more importantly, in both cases, the core variables were the same in all competing models, such that the importance of additional variables, and their influence on the overall model, could be clearly discerned.

## Discussion

Use of REVS with the considered datasets suggests that the technique provides much more useful results than typical regression approaches (full, stepwise, and all-subset models), particularly when the aim of the researcher is to identify the causal factors in a dataset and understand their effect on the dependent variable [5]. This is of fundamental importance when quantifying species-habitat relationships to inform management [11]. When analysing the case study dataset (habitat influence on great tit offspring quality), the best REVS model allowed identification of key variables, which the full model did not (indeed, the very presence of important predictors within the set of independent variables was masked by a high AIC, low R^2^ and overall non-significance). Only three variables were included in the stepwise model and these would not easily inform management. For example, it might appear from the stepwise results that poor-quality great tit chicks were associated with areas of high bracken coverage. However, when the REVS models are examined, it becomes apparent that actually they are associated with greater numbers of trees, particularly silver birch, which tend to occur in areas with less bracken (i.e. the multicollinearity of the dataset is masking the true picture). The fact that the best LEAPS and REVS models differed, and the low accuracy of the LEAPS model in predicting actual values of *Y* for cases in a hold-out sample, suggests that branch-and-bound techniques can compromise effectiveness for computational rapidity. Using LEAPS to quantify empirical support for each variable, and then generating new models based on this information in the way that REVS does, seems to be a rapid and convenient way to circumvent these problems.

Regression approaches are also common in other biological disciplines, including environmental biology and animal behaviour. Testing REVS on example datasets has demonstrated the wider applicability of the approach, with REVS models again being more parsimonious than full models, greater model fit (resulting in higher R^2^ values), and lower delta AIC values. When compared with stepwise regression, the best REVS model typically had a higher R^2^ value, a lower delta AIC value and lower P values. In 18% of datasets tested here, the best REVS and the best stepwise model were synonymous. This is not surprising given that both techniques are used to achieve the same end result – indeed it could be argued that if stepwise procedures were optimal, stepwise and REVS results should be synonymous. The same is true of comparing REVS with LEAPS all-subset models – in 60% of cases the models are identical, but in 40% of cases the REVS models are superior. Importantly, REVS was never inferior to stepwise or LEAPS all-subsets, so use of REVS appears to be, at worse, the same as these procedures and at best, improve upon them.

REVS does not rely on model building using a one-at-a-time method of adding/deleting variables since the empirical support for each variable is gained multivariately. Thus the “optimal” model should not be missed [14], [19] and the inclusion/exclusion of individual variables is likely to be biased [20], [21]. There is no reliance on p-values, such that there is no arbitrary cut-off point and artificial inflation of the significance of individual predictors and potential type II errors [14] are avoided. Given the way that ties in variable ranking are handled in REVS (see Methods), the order of the predictor variables in the dataset will also have no effect, unlike in many stepwise scenarios [24]. Finally, because AICs are used, multiple models can be considered and there is no reliance, implicit or explicit, on a MAM [5], [9], [16]. This also means that the researcher does not have to be confined by a potentially-unhelpful null hypothesis framework [28], although significance values can be generated and used if this is helpful; for example in lab studies where a specific hypothesis has been generated [9]. Interpretation of multiple competing models, in situations when reliance on a MAM is unsuitable, is easier for REVS than for LEAPS as there are fewer models to consider and more similarities between candidate models. In summary, REVS is a rapid and intuitive analytical method, with general applicability in biological/ecological correlative studies, that avoids the usual weaknesses of full-model and stepwise regression.

## Supporting Information

File S1 **R code for the REVS procedure (requires R to open; download R software package from** http://cran.r-project.org **if necessary and ensure that the LEAPS package is installed).**
(R) Click here for additional data file.

File S2 **Sample dataset for use the REVS procedure (CSV format).**
(CSV)

File S3 **A ReadMe document that provides a practical introduction to REVS.** It also describes how to run REVS on the sample data (File S2) and briefly interprets the results obtained by so-doing.
(DOC)
