# Supplementary material for: Regression with Empirical Variable Selection: Description of a New Method and Application to Ecological Datasets
Source: PLoS One. 2012 Mar 30;7(3):e34338. doi: 10.1371/journal.pone.0034338 (PMC3316704; doi:10.1371/journal.pone.0034338)
Supplement: File S3 — A ReadMe document that provides a practical introduction to REVS. It also describes how to run REVS on the sample data (File S2) and briefly interprets the results obtained by so-doing. (DOC) [file pone.0034338.s003.doc]

**ReadMe.file**

This file: (1) supplies details of the sample data file SampleData.CSV; (2) describes what happens when REVS is run on this data file; and (3) briefly interprets the results that are then obtained.

The details here are intended to supplement those given in the main paper by providing a practical introduction to REVS.

1. Details of SampleData.CSV

This file contains real field and lab data (unpublished) collected by Stewart Eley from the University of Gloucestershire as part of his undergraduate dissertation and are used here with his permission.

The data comprise one dependent variable (Column 1) and five candidate independent variables (Columns 2-6). There are 13 rows – a heading row and 12 data rows.

This data file has been chosen because it is simple (few variables) and short (few cases). It is thus quick to run, does not generate screeds of output, and is easy to comprehend, making it a perfect introductory file. The data are, however, complex enough that the basic premise of REVS is illustrated.

The dependent variable is the mean number of bacterial colony forming units cultured on nutrient agar plates from water collected from 12 different sites. The candidate independent variables are water chemical parameters from the same sites – dissolved oxygen, Biological Oxygen Demand (BOD), pH, nitrogen level and phosphorus level.

2. Running REVS on this file

When REVS is run, the order of the columns is changed to reflect the level of empirical support for each variable (see paper for more details), such that the independent variable with the most support is shuffled to Column 2, the one with the next most support is shuffled to Column 3 etc. The dependent variable always remains in the first column.

The output from REVS gives the new column order: the variable names (taken from the headings for each column that were originally typed in by the user in the CSV file) are reported in their new order. In this case, the original column order is replaced as detailed below:

Original Order: Oxygen BOD pH Nitrogen Phosphorous

New Order: Phosphorous pH Nitrogen Oxygen BOD

Thus, in this case, the variable with the most empirical support is “Phosphorous”.

Statistical output – AIC, delta AIC and adjusted R^2^ values – are reported for the best model at all Levels (see paper). In this case, because there are five variables, five values are reported for each parameter (the first relates to the model with just “Phosphorous” being entered, the second to the model with both “Phosphorous” and “pH” and so on).

Above this summary section, full details of the single best REVS model (the one with the lowest AIC) are given – this includes details of the P value, degrees of freedom and the unadjusted multiple R^2^, as well as the adjusted R^2^.

Below the output relating the REVS, the user might find details of stepwise models and the full models, if the option to uncomment this section of code to activate it (see below) has been taken. In this case, please note that the variables entered into specific stepwise models are referred to by their column number. This number relates to the REORDERED data file (see above). In the case of SampleData.CSV, if the variables listed in columns 2 and 5 are entered that denotes that Phosphorous and Dissolved Oxygen have been entered (remember that the dependent variable is always column 1)

3. Output Summary

When the REVS code is run (with the comparison to stepwise and full models facilitated by the final section of the REVS code being uncommented to activate it), the following results are obtained:

|  | Best REVS Model | Best Stepwise Model | Full Model |
| --- | --- | --- | --- |
| Predictors included* | Phosphorous (2)  pH (3) | Phosphorous (2)  Dissolved Oxygen (5) | Phosphorous (2)  pH (3)  Nitrogen (4)  Oxygen (5)  BOD (6) |
| AIC | 92.61 | 94.47 | 97.37 |
| Delta AIC | 0.00 | 1.86 | 4.76 |
| Adj R^2^ | 0.43 | 0.33 | 0.23 |
| P | 0.03 | 0.07 | 0.28 |

* = Numbers in parentheses denote the column number of each variable (the dependent variable is always column 1).

The difference in the AIC values (and thus the delta AIC) here is comparatively small given the reasonably simple data structure and few cases. The advantages of REVS are, however, clear. The R^2^ values are substantially higher and a significant result only occurs when using REVS.
